# The power of heteronemin in cancers

**DOI:** 10.1186/s12929-022-00816-z

**Published:** 2022-06-15

**Authors:** Kuan Wang, Yi-Fong Chen, Yu-Chen S. H. Yang, Haw-Ming Huang, Sheng-Yang Lee, Ya-Jung Shih, Zi-Lin Li, Jacqueline Whang-Peng, Hung-Yun Lin, Paul J. Davis

**Affiliations:** 1 Graduate Institute of Nanomedicine and Medical Engineering, College of Medical Engineering, Taipei Medical University, 250 Wuxing Street, Taipei 110, Taipei, 11031 Taiwan; 2 Graduate Institute of Cancer Molecular Biology and Drug Discovery, College of Medical Science and Technology, Taipei Medical University, Taipei, 11031 Taiwan; 3 Joint Biobank, Office of Human Research, Taipei Medical University, Taipei, 11031 Taiwan; 4 School of Dentistry, College of Oral Medicine, Taipei Medical University, Taipei, 11031 Taiwan; 5 Dentistry, Wan-Fang Medical Center, Taipei Medical University, Taipei, 11031 Taiwan; 6 Cancer Center, Wan Fang Hospital, Taipei Medical University, No. 111, Section 3, Xinglong Road, Wenshan District, Taipei City, 116, Taipei, 11031 Taiwan; 7 TMU Research Center of Cancer Translational Medicine, Taipei Medical University, Taipei, 11031 Taiwan; 8 Traditional Herbal Medicine Research Center of Taipei Medical University Hospital, Taipei Medical University, Taipei, 11031 Taiwan; 9 Pharmaceutical Research Institute, Albany College of Pharmacy and Health Sciences, Rensselaer, NY 12144 USA; 10 Department of Medicine, Albany Medical College, Albany, NY12144 USA

**Keywords:** Heteronemin, Anticancer, Sponge, Marine sesterterpenoids, Integrin αvβ3

## Abstract

Heteronemin (Haimian jing) is a sesterterpenoid-type natural marine product that is isolated from sponges and has anticancer properties. It inhibits cancer cell proliferation via different mechanisms, such as reactive oxygen species (ROS) production, cell cycle arrest, apoptosis as well as proliferative gene changes in various types of cancers. Recently, the novel structure and bioactivity evaluation of heteronemin has received extensive attention. Hormones control physiological activities regularly, however, they may also affect several abnormalities such as cancer. *L*-Thyroxine (T_4_), steroid hormones, and epidermal growth factor (EGF) up-regulate the accumulation of checkpoint programmed death-ligand 1 (PD-L1) and promote inflammation in cancer cells. Heteronemin suppresses *PD-L1* expression and reduces the PD-L1-induced proliferative effect. In the current review, we evaluated research and evidence regarding the antitumor effects of heteronemin and the antagonizing effects of non-peptide hormones and growth factors on heteronemin-induced anti-cancer properties and utilized computational molecular modeling to explain how these ligands interacted with the integrin αvβ3 receptors. On the other hand, thyroid hormone deaminated analogue, tetraiodothyroacetic acid (tetrac), modulates signal pathways and inhibits cancer growth and metastasis. The combination of heteronemin and tetrac derivatives has been demonstrated to compensate for anti-proliferation in cancer cells under different circumstances. Overall, this review outlines the potential of heteronemin in managing different types of cancers that may lead to its clinical development as an anticancer agent.

## Background

Cancers cause the highest mortality rate worldwide. Searching for cancer prevention and treatment is urgent. Marine secondary metabolites process several biological functions including anti-inflammatory, antimicrobial, antiviral, and antioxidant activities [1]. They also produce antimicrobial ichthyotoxin, protein inhibitory, and antimalarial activities [2, 3]. More importantly, those secondary metabolites exhibit anticancer properties [1–4]. The anti-cancer activity is the most attractive biological function induced by marine secondary metabolites-induced biological functions. Studies have shown the extraordinary anti-cancer potential of marine compounds targeting a variety of kinases in different types of cancers [5]. Furthermore, marine organisms’ secondary metabolites of marine compounds—such as alkaloids, terpenes, peptides, anthraquinones, and steroids—have potent anticancer activities [6]. Heteronemin (海綿精, Haimian jing) is the most plentiful secondary metabolite in the sponge *Hippospongia sp.*. Heteronemin and its semisynthetic derivatives express important cytotoxic activity against a variety of tumor cells [7–9]. Heteronemin also disrupts various signal transduction pathways and has endocrine hormone interactions; these interactions include steroids [10] and thyroid hormones [11, 12]. Additionally, the non-peptide hormones have been shown to interfere with the efficacy of anti-cancer drugs. For example, thyroxine (T_4_) diminishes anti-cancer activities induced by cetuximab [13] and gefitinib [14, 15]. T_4_ inhibits resveratrol-induced apoptosis by activating programmed death-ligand 1 (PD-L1) in ovarian cancer cells [16]. Estrogen blocks heteronemin-suppressed *PD-L1* expression and anti-proliferation [10]. Recently, heteronemin has attracted the attention of pharmacologists and chemists mainly because of its potential anticancer properties. The complex mechanisms underlying heteronemin’s inhibition of cancer growth in animals and in clinical studies are to be more extensively evaluated. Human prostate cancer xenografts are significantly reduced in size by heteronemin treatment of tumor-bearing mice without serious adverse effects, such as loss of body weight [17]. Similarly, the drug reduces tumor size in human leukemia xenograft-bearing mice [18]. Much more needs to be known about the molecular basis of heteronemin’s anticancer activity. Cellular uptake of the agent and actions of the drug on signal transduction pathways require further investigation, as do the mechanisms by which hormones and growth factors interfere with the heteronemin-induced anticancer activity [10–12, 19, 20]. The contributions of integrins in cancer progression have recently attracted attention in the literature [21, 22]. We have studied integrin αvβ3-linked signal transduction pathways and have demonstrated that integrin αvβ3 contains a receptor site for thyroid hormone analogues that permits *L*-thyroxine (T_4_), the principal secretory product of the thyroid gland, to induce cancer growth [23]. Importantly, we and others have shown that the deaminated thyroxine analogue, tetraiodothyroacetic acid (tetrac), and its nanoparticulate analogue, nano-diamino-tetrac; NDAT) compete with T_4_ for the thyroxine-binding site on the integrin αvβ3 to inhibit cancer cell growth [24]. Some studies have demonstrated that the combination of heteronemin and tetrac (or NDAT) can enhance the anticancer effects [11, 12]. In the current review, mechanisms of heteronemin-induced antiproliferation in cancer cells will be evaluated and discussed. Additionally, the effects of hormones and growth factors on heteronemin-induced mechanisms will be addressed. Taking together, we presume that the integrin αvβ3 is the novel target in cancer cells that mediates the antitumor activity of heteronemin and we apply molecular modeling to define the interaction of heteronemin and integrin αvβ3. In addition, the published efficacy of the principal tetrac-containing lead candidate (fb-PMT) shows high integrin αvβ3 binding potency with an IC_50_ of 0.23 nM against glioblastoma and acute myeloid leukemia [25, 26]. The animal studies found no recurrence or relapse of xenografts with discontinuation of fb-PMT and cancer cells have been replaced by normal cells [25]. Currently, the fb-PMT is successful to process in phase 1 clinical trial to evaluate its safety and tolerability. These results encourage scientists to discover other anticancer agents targeting integrin αvβ3, and the heteronemin or its derivatives may be the next cancer-curing star.

## Current incidence of cancers

The incidence of new cancers worldwide in 2020 was 19.3 million, with 10 million cancer-related deaths [27]. Globally, breast cancer remains the most common cancer, although specific cancer incidences vary. Triple-negative breast cancer remains an important therapeutic concern. In the developed western world—Northern and Western Europe, North American and Oceania—prostate cancers are ranked the sixth leading death cause of cancer in men [28]. The prognosis for prostate cancer can be severe, with more than 80% of patients with advanced prostate cancers likely to have bone metastases. More than 30,000 prostate cancer deaths result from drug resistance to the semi-synthetic natural product docetaxel, approved by the US Food and Drug Administration (FDA) as a first-line treatment [29, 30]. With the aging of the population, there is an urgent need to develop more effective and safer drugs which are needed as treatment for cancers of increased incidence in the elderly. In this regard, certain natural compounds, such as heteronemin, are attracting attention for investigation and development as cancer treatments.

## Characteristics of heteronemin

The area of oceans occupies more than 70% surface of the earth. Oceans’ complex ecosystems provide enormous biodiversity [31]. Certain groups of marine biomaterials offer medical utilities, especially in anti-cancer activities. Marine organisms’ secondary metabolites process special skeleton characteristics and various biological functions making them serve as treasures for lead drug development. Heteronemin, a member of scalarane sesterterpenoids, is the most plentiful secondary metabolite isolated from the marine sponge *Hyrtios sp*.. The atomic structure of heteronemin was first defined in 1976 [32] and its precise stereo-structure was determined by X-ray crystallographic analysis in 1991 [33]. Heteronemin has been shown to be importantly cytotoxic in a variety of cancer cells [34], but the agent has low or negligible cytotoxicity in normal cells [34–36].

**Fig. 1:**
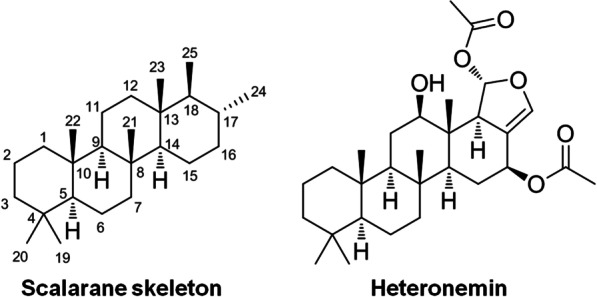
The structure of scalarane skeleton and heteronemin [40]

Heteronemin has been isolated from various sponge species including *Hyrtios erecta* [36–38], *Hippospongia sp.* [7], *Hyrtios reticulate* [39], and *Brachiaster sp*. [8]. The agent is a pentacyclic scalarane-type sesterterpene. Scalarane sesterterpenoids attract attention because of their diverse biological characteristics that allow them to modify the molecular backbone structure to create various potent derivatives [9, 40]. Heteronemin is an excellent example of this. Heteronemin contains a pentacyclic ring that includes dihydrofuran moiety (Fig. 1) [9]. The several functional groups of the compound’s complex structure include a pentacyclic scalarane skeleton with the dihydrofuran. There are nine chiral centers, two methyl groups at C-4, and a secondary hydroxy group at C-12. In addition, it contains an acetal moiety at C-25, an acetoxy group at C-16, another acetoxy group at C-25, and a double bond at C-17–24. The distinctively functional construction synthetically permits its structural modification and improves its biological activity. [9].

## Heteronemin induces anti-proliferation in cancer cells

As noted above, heteronemin displays potent anti-proliferation in several types of cancer cells, while exhibiting no action on nonmalignant cells such as oral gingival cells (HGF-1) and mucosa cells (OMF) [36]. Other studies by Cheng et al. showed that heteronemin was cytotoxic in lung cancer A549 cells with IC_50_ approximately 5.12 μM, in brain cancer GBM cells (IC_50_ approximately 7.12 μM) and U87 cells (IC_50_ approximately 9.58 μM), and in hepatoma HepG2 cells (IC_50_ approximately 12.55 μM) [36]. The antiproliferative activities of 12-oxoheteronemin and heteronemin were evaluated additionally in six cancer cell lines and IC_50_ values ranging from 0.66 to 1.35 µM were obtained [41]. Interestingly, heteronemin not only showed a strong potency in two estrogen receptor (ER)-positive breast cancer cells (MCF-7 and T-47D), but also exerted a notable antiproliferation in two ER-negative cell lines (MDA-MB-231 and Hs578T) [10, 41]. Thus, heteronemin may both interfere with estrogenic steroid hormones and exhibit anticancer activity through other pathways without steroid receptors. The summarized IC_50_ of heteronemin-induced anti-proliferation in cancers is listed (Table 1). The antiproliferative IC_50_ values of heteronemin range from micromolar (almost less than 10 μM) to nanomolar (less than 1 μM) concentrations. These results suggest that heteronemin has the potential for cancer therapeutic effects.

**Table 1:** The IC_50_ value of heteronemin-induced anti-proliferation in cancers

Cancer cell types	IC_50_.^a^ (Cell Lines)	Researchers	References
Brain cancer	7.12 μM (GBM) 9.58 μM (U87)	Cheng, M.H. et al.	[36]
Leukemia	approximate 2.8 μM (K562)	Schumacher, M. et al.	[42]
	0.40 μg/mL (K562)	Chen, Y.C. et al.	[18]
	0.16 μg/mL (HL60)	Chen, Y.C. et al.	[18]
	0.11 μg/mL (Molt4)	Chen, Y.C. et al.	[18]
	0.001 μM (K562)	Chang, Y.C. et al.	[7]
Melanoma	15.3 μM (SK-MEL)	Kamel, H.N. et al.	[9]
Oral cancer	0.37 μM (KB)	Wonganuchitmeta, S.N. et al.	[8]
Breast cancer	0.001 μM (T-47D)	Chang, Y.C. et al.	[7]
	0.8672 μM (MDA-MB-231) 0.8779 μM (MCF-7)	Yang, Y.S.H., et al.	[10]
	0.29 μM (MCF-7)	Wonganuchitmeta, S.N. et al.	[8]
	11.2 μM (BT549)	Kamel, H.N. et al.	[9]
	0.66 μM (MDA-MB-231) 0.65 μM (Hs578T) 0.70 μM (MCF-7) 0.77 μM (T47D)	Kittiwisut, S. et al.	[41]
Non-small cell lung cancer (NSCLC)	0.51 μM (A549) 0.48 μM (H1299)	Chung, C.C. et al.	[12]
	5.12 μM (A549)	Cheng, M.H. et al.	[36]
	14 μM (A549)	Alarif, W.M. et al.	[37]
Hepatocellular Carcinoma	12.55 μM (HepG2)	Cheng, M.H. et al.	[36]
	20 μM (HepG2)	Alarif, W.M. et al.	[37]
	10.4 μM (HA22T) 5.25 μM (HA59T)	Chang, W.T. et al.	[43]
Cholangiocarcinoma	4.4 μM (HuccT1) 3.9 μM (SSP-25)	Lin, H.Y. et al.	[44]
Renal carcinoma	1.57 μM (A498) 3.54 μM (ACHN)	Wu, S.Y. et al.	[38]
Colon cancer	0.001 μM (DLD-1 and HCT-116)	Chang, Y.C. et al.	[7]
	0.39 μM (HT-29)	Wonganuchitmeta, S.N. et al.	[8]
Cervical carcinoma	0.45 μM (HeLa)	Wonganuchitmeta, S.N. et al.	[8]
	0.82 μM (HeLa)	Kittiwisut, S. et al.	[41]
Ovarian cancer	3.4 μM (SKOV3)	Kamel, H.N. et al.	[9]
Prostate cancer	2.38 μg/mL (LNCap) 1.65 μg/mL (DU145) 6.11 μg/mL (PC-3)	Wu, J.C. et al.	[19]
	1.4 μM (LNCap) 2.7 µM (PC-3)	Lee, M.G. et al.	[17]
	35 µM (PC-3)	Alarif, W.M. et al.	[37]
	0.66 µM (PC-3)	Kittiwisut, S. et al.	[41]

^a^The IC_50_ value is represented as half-maximal inhibitory concentration

Heteronemin suppresses cancer growth in various types of cancers via multiple, diverse mechanisms. Heteronemin effectively restrains anchorage-independent growth and the viability and human prostate cancer cells [19]. Heteronemin inhibits the signal pathway-dependent proliferation [19, 44] and colony formation of refractory prostate cancer cells [19]. In addition, it also regulates cell adhesion, the expression of extracellular matrix (ECM) receptors, cell motility, the integral membrane, metastasis response, matrix metalloproteinase (MMP) remodeling, regulation of metabolism, sprouting angiogenesis, transcription factor, and vasculogenesis in cholangiocarcinoma cell lines. Heteronemin suppresses transforming growth factor-beta (TGF-β) expression with inhibition of growth, migration, and adhesion effects concurrently [44]. In addition, it also modulates different signal transduction pathways. Schumacher et al. pointed out that heteronemin down-regulates tumor necrosis factor alpha (TNFα)-induced nuclear factor kappa B (NF-κB) activation via proteasome [42]. Programmed cell death (apoptosis) [45] is another action of certain anticancer pharmaceuticals. Importantly, heteronemin also induces apoptosis to prevent TNFα-induced cell proliferation [42]. In addition, heteronemin activates apoptosis by the activation of both intrinsic (caspase-9) and extrinsic (caspase-8) apoptosis pathways in prostate cancer cells [19].

### Heteronemin reduces tumor size in human cancer in vivo xenograft animal models

Lee, M. G. and their co-workers have demonstrated that intraperitoneal injection (1 mg/kg) of heteronemin can significantly reduce tumor size compared to a control group in human prostate cancer (LNCap) xenograft animal model [17]. After a period of 29 days, tumor size in the control group increased to 76.1%, whereas tumor size in the heteronemin-treated group decreased to -15.8%. More importantly, the body weight of the mice did not show a significant difference between the control and heteronemin-treated groups throughout this animal study. These results showed that heteronemin not only displayed a cytostatic effect but also exhibited cytotoxicity against cancer cells, however, it was not harmful to normal cells. On the other hand, heteronemin also completely suppressed tumor growth in human leukemia (molt4) in vivo xenograft animal model, whereas the control group showed a 60% increase in tumor volume [18]. Additionally, plasma profiles of GOT, GPT, BUN, CRE and UA are not alternated by intraperitoneal administration of heteronemin (0.31 μg/g). These results illustrated that the tumor size was extremely reduced by heteronemin in vivo without any liver and kidney side effects.

### Heteronemin affects cell cycle progression

Cell cycle arrest can play a vital role in inhibiting tumor cell growth [46–48]. Treatment of human lymphatic endothelial cells with heteronemin increases the percentages of the G0/G1 phase significantly. Furthermore, the percentage of cells in the S- and G2/M phase was significantly reduced in heteronemin-treated lymphatic endothelial cells, suggesting heteronemin induces G0/G1 arrest in human lymphatic endothelial cells [20]. Heteronemin at 2.5 and 5 µM alters the distribution of cell populations at the sub-G1 phase in apoptotic HeLa cells [41]. Different concentrations of heteronemin altered different cell-cycle stages, and the accumulation of cells in G1 and G2/M was increased by heteronemin combined with tetraiodothyroacetic acid (tetrac) in OEC-M1 and SCC-25 cells, respectively [11]. Alternatively, heteronemin increased the cell population accumulations at the cell cycle G0/G1 phase compared to control of both non-small cell lung cancer, A549 and H1299 cells [12]. Interestingly, heteronemin only increased the sub-G1 phase in H1299 cells suggesting that apoptosis was involved. Both 12-oxoheteronemin and heteronemin increased sub-G1 populations of cells and caspase-dependent PARP cleavage to initiate apoptosis rapidly but did not affect cell cycle distribution in HeLa [41]. Topoisomerase II links with DNA replication [49]. Several types of clinical anticancer drugs, such as topotecan and irinotecan are inhibitors of topoisomerase II and topoisomerase I [50, 51]. Heteronemin inhibits topoisomerase II activity as well as Hsp90 functions [17]. In addition, heteronemin alters the binding of trans-activation response DNA-binding protein of 43 kDa (TDP-43)-cognate nucleic acids [52]. These results show that heteronemin interferes with DNA-binding DNA replication and cell cycle process and may have sufficient potential as an anticancer agent.

### Heteronemin induces ROS production

Cancer cells alter metabolic pathways to facilitate increased proliferation and cell survival resulting in glucose and glutamine addiction. The production of reactive oxygen species (ROS) is increased during cancer cell proliferation. Importantly, ROS production from mitochondria may also increase in response to heteronemin [18], but this serves to support ROS-dependent apoptosis. ROS includes hydroxyl radical (OH^•^), hydrogen peroxide (H_2_O_2_), and superoxide (O_2_^•–^) [53]. Both aerobic glycolysis and mitochondrial oxidative phosphorylation are cellular sources of ROS. At low concentrations, ROS is an essential signaling molecule, however, high quantities of ROS impair macromolecules such as DNA, triggering senescence [54]. Heteronemin may permeabilize mitochondria leading to cytochrome c release and induction of apoptosis [53, 55, 56]. Mitochondria have been shown to be involved in major pathways for apoptosis [57]. B-Cell Lymphoma 2 (Bcl-2) family proteins highly modulate mitochondrial-mediate apoptosis. Bcl-2 family proteins comprise both anti-apoptotic (Bcl-2 and Bcl-xL) and proapoptotic (Bax and Bak) members [58]. The balance between the expression levels of pro-and anti-apoptotic proteins is critical for cell survival or cell death [57]. On the other hand, ROS is also linked with the mitogen-activated protein kinase (MAPK) pathway and NF-κB increases [59].

Several types of ROS such as superoxide anions (O_2_^•–^), hydrogen peroxide (H_2_O_2_), and hydroxyl radicals (OH^•^), have been shown to play a vital role in chemotherapy. They mediate several cellular pathways related to apoptosis and ferroptosis that involve cell fates. Heteronemin is able to induce ROS accumulation to promote oxidative stress directly and cytotoxic effects [18, 43]. Heteronemin-induced ROS is associated with heteronemin-induced cell death. Mitochondrial superoxide dismutase 2 (SOD2) rather than cytosolic SOD1 triggers ROS removal in hepatocellular carcinoma [43]. The dismissed ROS may hamper apoptosis in cancer cells. Heteronemin-induced ROS production and apoptosis have been demonstrated in different types of cancer cells [17, 18, 43]. On the other hand, heteronemin can also induce ROS-independent apoptosis in cancer cells [43]. Alternatively, heteronemin suppressed p53 activity in cancer cells [44] and suppressed TGF-β-dependent cell proliferation [44]. In addition, MAPK signaling pathway also plays important role in ROS-induced cell death. Heteronemin controls the Bcl-2 family mediated apoptotic pathway [17, 38] and autophagy [38]. Heteronemin increases talin phosphorylation in both Molt4 cells and human embryonic kidney 293 (HEK293) cells but only talin expression in Molt4 cells [18]. N-acetyl cysteine (NAC), a ROS scavenger, inhibits heteronemin-induced talin activation [18]. Consequently, ROS-induced phosphorylated talin expression results in cell apoptosis. Furthermore, heteronemin interferes with actin microfilament and this may lead to morphologic changes [18].

Recently, a unique non-apoptotic programmed cell death called ferroptosis [43] which is iron-dependent cell death has been described. Ferroptosis is associated with ROS and lipid peroxides that have been found to activate inflammation and induce cell death. Ferroptosis motivates the effect of many first-line chemotherapeutic drugs such as cisplatin [60] and sorafenib [61] for advanced cholangiocarcinoma [62] and hepatocellular carcinoma (HCC) [63]. Heteronemin induces both ferroptosis and apoptosis in HCC [43]. In contrast, treatment with a ferroptosis inhibitor is also able to restore heteronemin-induced cell death. The anticancer effect of heteronemin on HCC is associated with ROS-associated MAPK activation. Heteronemin inhibits glutathione peroxidase 4 (Gpx4) expression that suppresses ferroptosis [43, 64]. Thus, with appropriate structural modification, heteronemin can act as a potent therapeutic agent against HCC [43].

### Heteronemin regulates signal transduction pathways

Heteronemin has been shown to bind to vascular endothelial growth factor receptor-3 (VEGFR-3) and reduce downstream phosphorylation of mitogen-activated protein kinase (MAPK)/extracellular signal-regulated kinase (ERK) and nuclear factor-κB (NF-κB) [20]. However, the interaction mechanism has not been fully investigated. After uptake, most likely by endocytosis, heteronemin modulates different signal transduction pathways. As a farnesyl transferase inhibitor (FTI), heteronemin can inhibit both cytarabine-induced and farnesyl transferase-dependent RAS activation [65]. Heteronemin down-regulates activation of RAS, thus it sequentially modulates different signal transduction pathways including MAPK, activator protein-1 (AP-1), NF-κB, and c-Myc [65]. In addition to modulating signal transduction, heteronemin can modify actin microfilament and induce shape changes [18], as noted above; such morphologic changes may alter the physical access of components of signal transduction pathways to one another. Heteronemin can also induce cytotoxic effects via oxidative stress and the increasing accumulation of phosphorylated talin [18]. However, heteronemin does not disrupt talin/focal adhesion kinase (FAK) complex formation [44].

Suppressed activation of signal transducer and activator of transcription 3 (STAT3) and ERK1/2 is a vital process in antiproliferation in cancer cells [11, 24, 66]. Heteronemin inhibits activation of ERK1/2, and phosphoinositide 3-kinase (PI3K) [11] and their effector signals, for example, STAT3, with consequent changes in expression of STAT3-regulated genes, such as Bcl-xL, Bcl-2, and cyclin D1 [44]. The major MAPKs—such as extracellular signal-regulated kinases (ERKs), c-Jun N-terminal kinases (JNKs), and p38—respond to stimulatory inputs via alterations in their control of cell differentiation [67], cell cycle arrest [68], cell survival [69] and the inflammatory process [70]. Heteronemin inhibits the activation and expression of ERK1/2, thereby hampering cell growth [43]. Heteronemin increases the phosphorylation of p38 and JNK; however, cotreatment with inhibitors of JNK and p38 reverse heteronemin-induced cytotoxicity and apoptotic signaling [38, 43]. These results suggest that heteronemin produces ROS formation and induces apoptosis via the JNK/p38 multi-MAPK signaling pathway.

Cholangiocarcinoma is a malignant tumor of the biliary tract and the most common type of primary liver cancer [71]; the incidence of this type of cancer is increasing throughout the world. Effective chemotherapy for this tumor is not available [71, 72]. Cholangiocarcinoma is not easy to detect early and surgical resection is the only means of treatment. This form of cancer is resistant to most chemotherapeutic agents and commonly has a high mortality rate. Cisplatin or gemcitabine is the standard chemotherapeutic approach for cholangiocarcinoma [73, 74]. Several gemcitabine-resistant cell lines are also cross-resistant to 5-fluorouracil (5-FU), doxorubicin, and paclitaxel, indicating their multidrug-resistant nature [75]. Gefitinib, an epidermal growth factor receptor (EGFR) tyrosine kinase inhibitor [15, 76, 77] to enhance radiosensitivity in cholangiocarcinoma cells [71, 72]. However, several types of chemotherapeutic resistances have developed in gefitinib-treated tumors [78–80]. Different strategies have been applied to overcome chemotherapy resistance in *KRAS* mutant cancers [81]. Curcumin has been shown to induce autophagy-related cell death, overcoming primary gefitinib resistance in non-small-cell lung cancer (NSCLC) cells [80]. Loperamide has been used to trigger autophagy-independent apoptosis in gefitinib-resistant *KRAS* mutant NSCLC cells [82]. Lysine deacetylases (KDAC) inhibition suppresses cancer growth in mutated adenocarcinoma cells overexpressing amphiregulin via overcoming primary gefitinib resistant *KRAS* mutation [83]. Research indicates that *KRAS* status plays a key role in gefitinib resistance. Heteronemin downregulates both Ras and NF-κB signaling pathways [65]. Thus, the drug appears to have the potential as a targeting therapeutic agent to overcome the resistance status of EGFR tyrosine kinase inhibitors.

## Heteronemin regulates gene expression

Heteronemin has been shown to target trans-activation response DNA-binding protein of 43 kDa (TDP-43) [52]. Heteronemin modulates the TDP-43 aggregated state and the cellular localization of the protein; heteronemin also influences the binding of TDP-43-cognate nucleic acids [52]. This evidence indicates that this anticancer natural product may be capable of modulating gene expression directly or indirectly.

Heteronemin increases talin phosphorylation in both Molt4 cells and human embryonic kidney 293 (HEK293) cells but affects the expression of talin only in Molt4 cells [18]. Heteronemin inhibits TGF-β expression as well as cell proliferation, migration, and adhesion effects in cholangiocarcinoma [44]. The sponge product suppresses the expression levels of TGF-β, SMAD (mothers against decapentaplegic homolog), and Myc [44]. Heteronemin also modulates the expression of genes linked to signal transduction pathways in cancer progression, such as cell motility-linked genes in cholangiocarcinoma cell lines (HuccT1 and SSP-25 cells) and colorectal cancer HCT-116 cells [44]. Heteronemin inhibits ERK1/2 activation and expression, as indicated earlier in this review, the drug is therefore a down-regulator of cell growth [43].

Interestingly, heteronemin suppresses *p53* expression and activity in cholangiocarcinoma [44] and oral cancer cells [11], suggesting that heteronemin does not induce anti-proliferation via a p53-dependent pathway. The cytotoxic effect of heteronemin is associated with oxidative stress and induction of phosphorylated talin expression [18]. The sponge product also inhibits the expression of proliferation-related genes such as *CCND1*, thrombospondin-1 (*THBS-1*), *TGF-β1*, *PCNA*, *c-Myc*, and *PD-L1* in several types of cancers [10–12, 44]. Importantly, heteronemin also induces pro-apoptotic *p21* gene expression in a concentration-dependent manner. Heteronemin significantly suppressed the expression of *THBS-1* and *p53* at 0.313 µM in oral cancer [11]. Heteronemin and combined treatment with tetrac induce downregulation of *MMP-9* mRNA expressions in oral [11] and lung cancer cells [12]. Matrix metalloproteinases (MMPs) drive cell migration to promote tumor cell spread and metastasis [84]. Heteronemin suppresses the expression of PCNA in oral cancer [11] and also reduces EGFR, and PD-L1 significantly in breast [10] and lung cancer [12]. Whereas at 0.625 µM, heteronemin did not affect *p21* expression, it did suppress the expression of *c-Myc*, *EGFR*, and *PD-L1* in ER-negative MDA-MB-231 cells [10]. Immune checkpoint PD-L1 supports cancer cell growth [15]. Overexpression of PD-L1 was more frequent in RAS-mutated cells than in *RAS*-wild-type lung cancer cells [85]. As shown in Table 2, heteronemin regulates expressions of critical genes involved in cancer progression.

**Table 2:** The heteronemin-regulated gene expressions in cancer

Cancer Type	Activation	Inhibition	References
Oral cancer			
OEC-M1 cells	*p21*	*p53, CCND1, PCNA, TGF-* *β1*, *MMP-9* and *THBS-1*	[11]
SCC-25 cells	*p21* and *TGF-β1*	*p53, CCND1, PCNA*, *MMP-9* and *THBS-1*	
Breast cancer			
MCF-7 cells	*c-Myc*, *Bcl-2*, and *p21*	*ki-67*, *CCND1, UCP2*, *EGFR* and *PD-L1*	[10]
MDA-MD-231 cells	*p21*	*ki-67*, *CCND1*, *c-Myc*, *Bcl-2, EGFR* and *PD-L1*	
Non-small cell lung cancer (NSCLC)			
A459 cells	*P21, MMP-9*	*CCND1*, *CASP-2*, *p53,* and *PCNA*	[12]
H1299 cells	–	CCND1*, CASP-2*, *MMP-9, p53, PCNA* and *p21*	
Cholangiocarcinoma			
HuccT1 cells and SSP-25 cells	*HSPB1, HMOX1, SNAI1, SERPINH1, CREBBP, COL7A1, JUN, CLDN4, NDRG1, EIF2AK3, IL11, BTG1, NOTCH1, FGFR1, VEGFA, COL6A2, CAMK2D, HKDC1, SIRT1, NFAT5, DST, LAMA5, CD44, LTBP4, HSP90B1, SETD2, ZEB2, ADAM17, CDKN1A, ADM2, PLEKHO1, MYC, HIPK1, PLXND1, SERINC5, ADAMTS1, PLAUR, SRPK2, MMP1, ACHE* and *PFKFB4*	*ENPEP, IL1A, ITGB2, SNRPF, NRP2, CDC42, CD24, NRP1, PTTG1, RAC2, HPSE, RORB, ITGB6, EPHB4, GPR124, LAMC1, MYLK, TMEM30B, CLIC4, P3H2, SPOCK3, EIF4E2, ENO1, FREM2, CCDC80, CHI3L1, PXDN, CDH2, RBL1, ICAM1, ARHGDIB, RBM47, CALD1, NME1, MMP17, RB1, SDC4, RUNX1T1, CGN, PKM, TACSTD2, SACS, ITGA3, HK2, ALOX5, RBL2, GLYR1, STAT3, SRF, IL13RA2, DEN, COL6A1, CDS1, IL1B, FSTL1, MGAT5, CHD4, SMC3, EPHA1, CEACAM1, HOXB3, VAV3, ILK, C3, SLC2A1, BMP5, DICER1, EPHA2, CAV1, LDHA, KDM1A, IGFBP4, GALNT7, AHNAK, LRG1, VWA2, NFKB1, GTF2I, TP53, DAG1, CMA1, ITGA6, TFDP1, ITGB8, PDGFC, KCNJ8, CDH11, EDN1, PLS1, F3, SMAD3, THBS1, TGFBR2, PTX3, BMP4, VCAN, TGFB2* and *LAMA3*	[44]
Prostate cancer cells			
DU145 cells and PC-3 cells	–	*Bcl-xL*, *Bcl-2*, and *cyclin D1*	[19]

## The interactions between carcinogenic effectors and heteronemin

The plasma membrane-receptors, such as integrin αvβ3, estrogen receptor (ER), and epidermal growth factor receptor (EGFR), drive cancer cell growth. Current evidence suggests that ligands of steroids and growth factors for membrane receptors interact with integrin αvβ3 [86–89]. Heteronemin-induced anti-proliferation was partially reversed by estradiol in breast cancer cells [10]. On the other hand, heteronemin-induced anticancer effects are enhanced by combining with integrin αvβ3 antagonist in oral cancer [11] and lung cancer [12]. Consequently, we introduce the concept of heteronemin interactions in the modulation of thyroxine, estrogen, and epidermal growth factor with integrin αvβ3.

### Molecular docking modeling of heteronemin inside the cyclic RGD domain pocket of integrin αvβ3

**Fig. 2:**
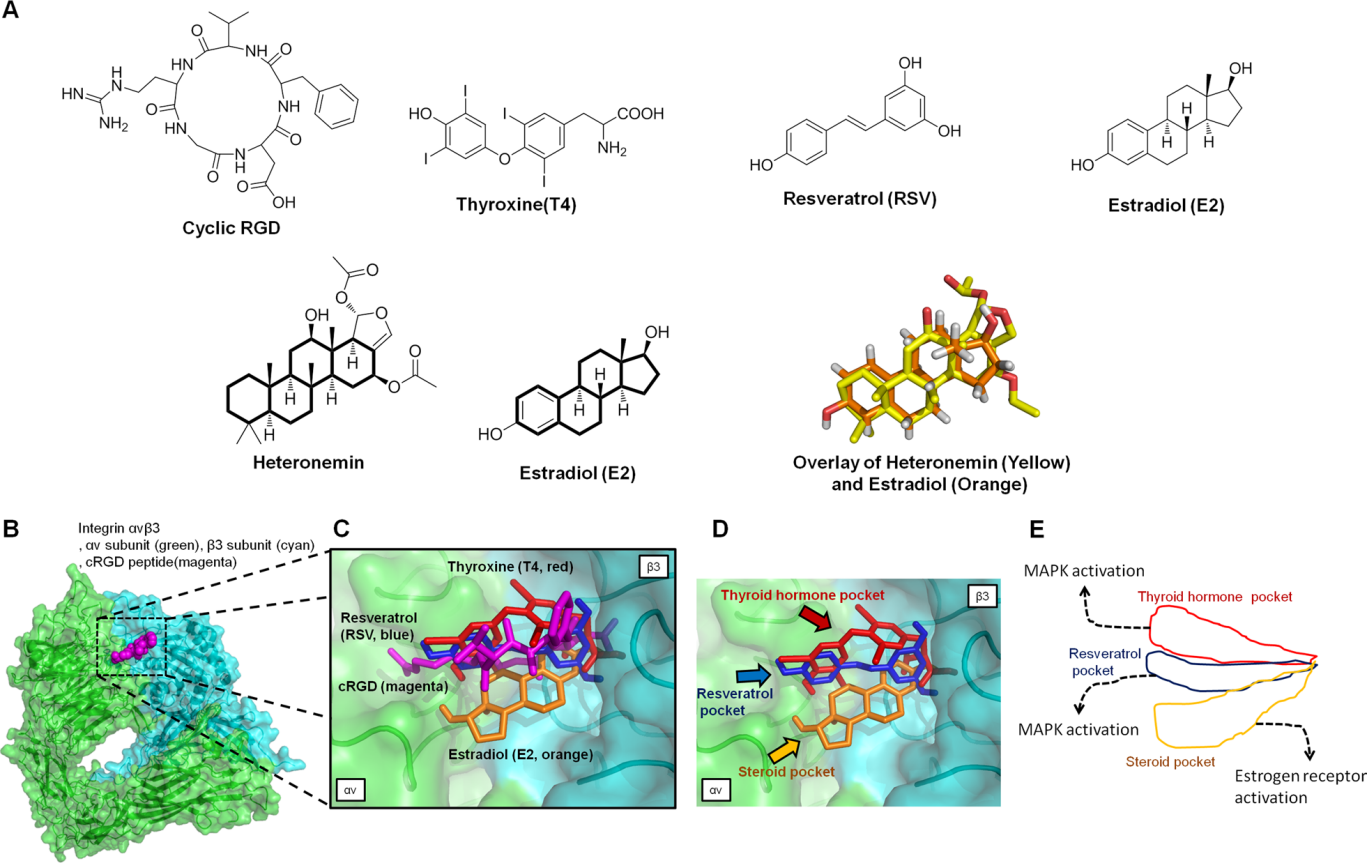
The backbone structure of heteronemin and estradiol and the docked binding modes of several ligands are shown with the RGD-recognition site of integrin αvβ3 (PDB entry 1L5G) [95]. Docking was carried out by AutoDock Vina software [96] in the RGD domain pocket. The grid map function in Auto-Dock 4.0 was used to define the interaction space of protein and ligand in the binding pocket. For ligand binding to the RGD domain site, a grid box of size 45 × 45 × 45 points was established in the x, y, and z directions, with the grid centers set at x = 19, y = 44, and z = 44. The docking pose results of the ligands were prepared and visualized with the graphic PyMOL (v. 1.3) program. **A** The chemical structures of integrin αvβ3 ligand include cyclic RGD (cRGD), thyroxine (T_4_), resveratrol (RSV), and estradiol (E_2_). Heteronemin (yellow) and estradiol (orange) show similar skeletons as presented in the black bold bond and three-dimensional superimposition. **B** The crystal structure of the integrin αvβ3 and cRGD complex is shown on the protein surface. Integrin αv subunit (green), β3 subunit (cyan), and cRGD peptide (magenta) are indicated by color. **C** Thyroxine (T_4_), resveratrol (RSV), and estradiol (E_2_) are performed in red, blue, and orange, respectively. The superimposition shows similar orientations of the binding site between the cRGD (magenta stick) and resveratrol (blue stick). **D** Predicted bound conformation of T_4_ (red), RSV (blue) and E_2_ (orange). The projections are according to previous publications [23, 90]. **E** According to the previous publication [23], the schematic representation is shown in three major pockets, including the thyroid hormone pocket, the resveratrol pocket, and the steroid pocket

As shown in Fig. 2A, several ligands are known to bind to integrin αvβ3 to activate biological functions [23, 90]. Previous reports indicated that sesterterpenoids have steroid-like structures [91]. Heteronemin is one of the scalarane-type marine sesterterpenoids, which are characterized by the typical tetracyclic carbon skeleton similar to that of estradiol (E_2_) steroids (Fig. 2A). Chen et al. discovered that the agent-containing tetracyclic-ring, such as doxycycline may also bind to integrin αvβ3 and interfere with downstream signaling [92]. Additionally, our groups have shown that E_2_ can bind to integrin αvβ3 to activate ERK1/2 [93]. The functional mechanism may require collaboration with the estrogen receptor-α (ER-α) [94]. In addition, thyroxine and E_2_ work on a similar pattern to induce nuclear co-localization of integrin αv and ER-α [94]. To investigate the possibility of heteronemin molecules’ interaction with integrins, we conducted modeling studies using the coordinates of the extracellular fragment of integrin αvβ3. In 2002, Xiong et al. utilized the X-ray methodology to resolve the crystal structure of the extracellular segment of integrin αvβ3 (PDB entry 1L5G) [95]. As shown in Fig. 2B, the crystal structure includes an arginine-glycine-aspartate (RGD) recognition site with an antagonist cyclic RGD (cRGD). In 2007, Cody et al. conducted computer molecular modeling of RGD recognition site on integrin αvβ3 with several small-molecule ligands, including L-thyroxine (T_4_), the active thyroxine metabolites tetraiodothyroacetic acid (tetrac, T_4_ac), resveratrol (RSV), and estradiol (E_2_) [90]. In this review, we also used computational molecular modeling to explain how these ligands interacted with integrin, and the docking data corresponded with previous results (our results are shown in Fig. 2C). In another report that was published in 2013, Davis et al. illustrated that the RGD binding domain of the integrin has three localized binding domains [23] (Fig. 2D, E): the thyroid hormone pocket (docking T_4_ and its antagonist T_4_ac, shown in red), the resveratrol pocket (docking resveratrol, shown in blue) and the steroid pocket (docking E_2_, shown in orange), respectively. In our analysis, the docking site of cRGD molecules overlaps the resveratrol-like pocket (Fig. 2C).

**Fig. 3:**
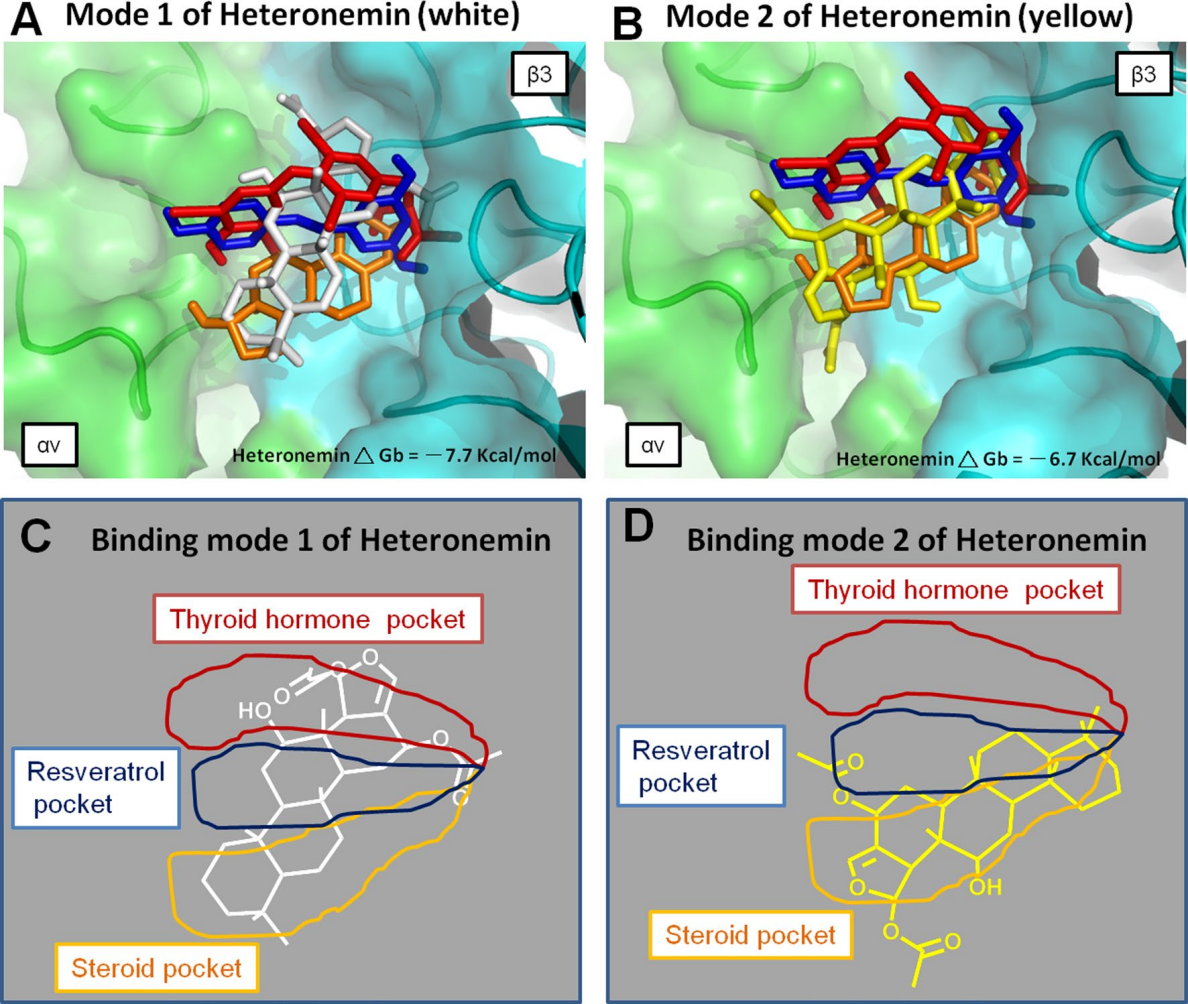
Crystal structure of integrin αvβ3 (PDB code 1L5G) [95] with thyroxine (T_4_, red), resveratrol (RSV, blue), estradiol (E_2_, orange), and heteronemin (mode 1 shown in white and mode 2 shown in yellow) models into the RGD binding pocket. The green and cyan parts represent αv and β3 chains of integrin αvβ3 receptor. **A** Docking mode 1 (white stick) of the heteronemin maps to T_4_ (red), RSV (blue), and E_2_ (orange). **B** Docking mode 2 (yellow stick) of the heteronemin maps to T_4_ (red), RSV (blue), and E_2_ (orange). **C** Schematic representation of observed interactions between binding mode 1 (white) of heteronemin and pocket sites. **D** Schematic binding mode 2 (yellow) of heteronemin

Since the heteronemin molecule has a steroid-like backbone, we speculate that the heteronemin also binds into a steroid pocket similar to estradiol. Interestingly, the docking data show two binding modes of heteronemin inside the cRGD domain pocket of integrin αvβ3 (Fig. 3A, B). One docked conformation fits into all of the thyroid, resveratrol, and steroid pockets, representing the binding mode 1 (Fig. 3A and C), and another is our projection that fits within the steroid pocket, representing binding mode 2 (Fig. 3B and D).

**Fig. 4:**
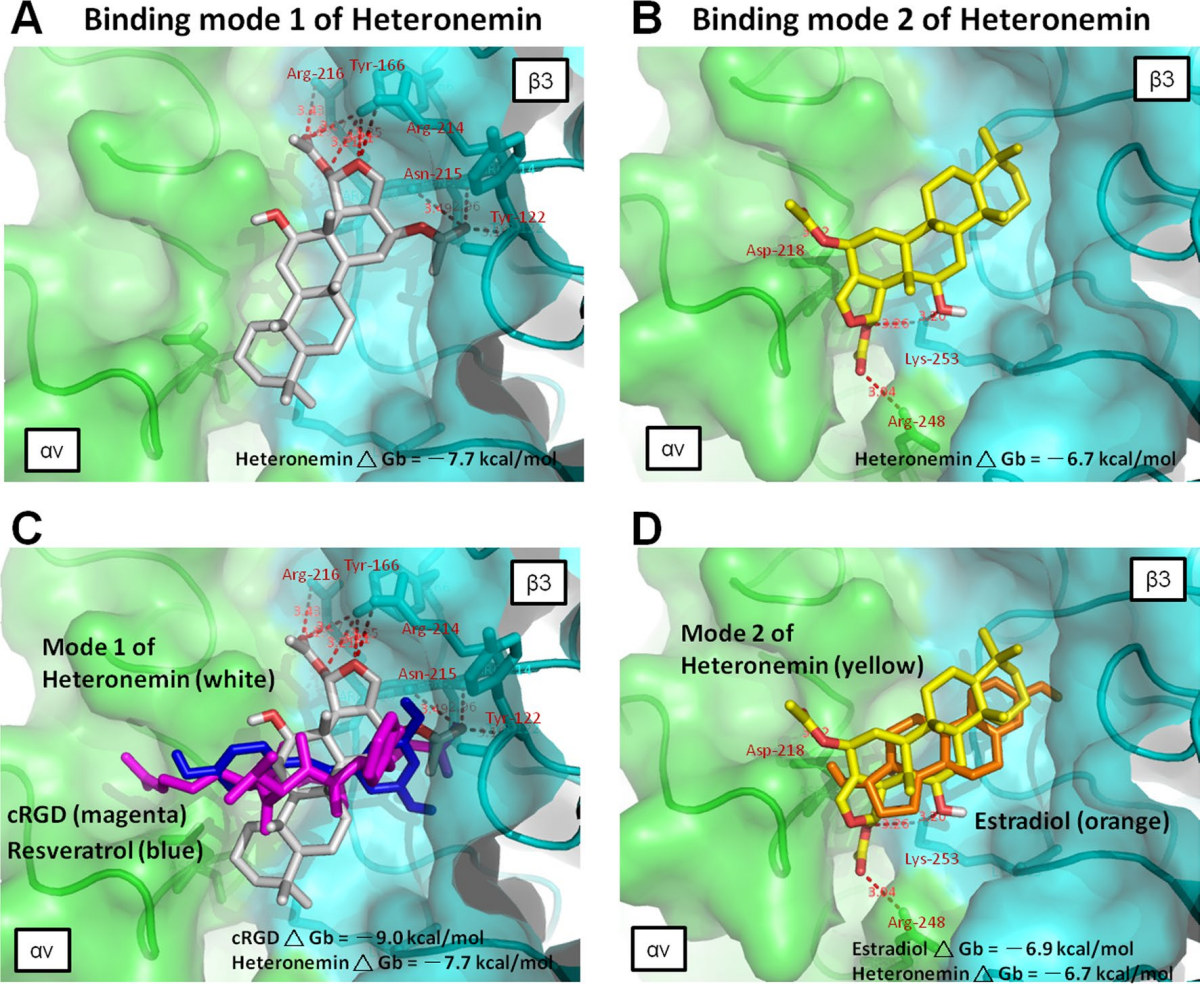
Molecular docking analysis of heteronemin on integrin αvβ3. **A** Heteronemin docking pose for mode 1 (white) in the RGD pocket is shown as a stick model. **B** The mode 2 of heteronemin (yellow) inside the RGD pocket of integrin αvβ3. **C** Protein–ligand interactions for heteronemin binding mode 1 (white stick) are analyzed to find hydrogen bonds displayed as red dashed lines. **D** Hydrogen bonds are displayed as red dashed lines in the heteronemin binding mode 2 (yellow stick)

The current review is the first report to propose, based on structural analysis, that the naturally occurring small molecule heteronemin utilizes the integrin αvβ3 to initiate biological functions. The modeling data indicate that heteronemin molecule fits the thyroid hormone pocket, resveratrol pocket, and the steroid pocket of integrins with minimal protein binding free energy (− 7.7 kcal/mol) (Fig. 4A), rather than docking only in the steroid pocket (− 6.7 kcal/mol) (Fig. 4B). Analytical views of mode1 and mode 2 interactions of heteronemin and integrin αvβ3 are shown in Fig. 4. In Fig. 4C, the cross-pocket pattern of heteronemin (white stick) indicated that the lone pair of electrons located in the dihydrofuran moiety of heteronemin formed hydrogen bonds with Arg-214 and Tyr-166; furthermore, the two acetate ester moieties formed hydrogen bonds with Arg-216, Asn-215, Arg-214, Tyr-166, and Tyr-122. In Fig. 4D, mode 2 of heteronemin (yellow stick) occupied a region of estradiol space close to the steroid pocket, and an acetate ester moiety of C-25 formed hydrogen bonds with Arg-248 and Lys-253, and another acetate ester moiety of C-16 formed a hydrogen bond with Asp-218; furthermore, the secondary hydroxy group of C-12 formed a hydrogen bond with Lys-253. As shown in Fig. 4C, heteronemin prefers to cross the resveratrol pocket binding site with a more stable binding conformation (lower free energy) because that formed more hydrogen bonds and is oriented perpendicular to the cRGD and resveratrol molecule. Collectively, this perspective provides insights into investigations of heteronemin for further targets with its anticancer efficacy.

### Heteronemin interacts with thyroid hormones

**Fig. 5:**
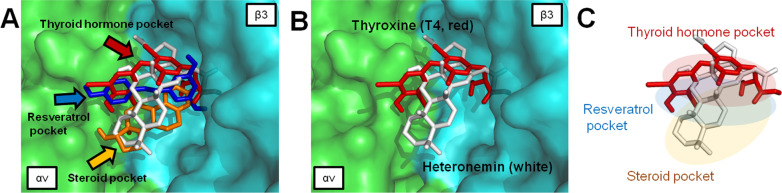
The best binding mode of heteronemin is superimposed with thyroxine. Thyroxine (T_4_), resveratrol (RSV), estradiol (E_2_), and heteronemin bind into integrin αvβ3, and were performed in red, blue, orange, and white, respectively. **A** T_4_, RSV, and E_2_ binding mode represent the thyroid hormone pocket, resveratrol pocket, and steroid pocket, respectively. **B** The best-docked mode of heteronemin maps with the T_4_. **C** Heteronemin prefers to occupy the binding sites across the thyroid hormone pocket

Thyroid hormones, triiodothyronine (T_3_) and thyroxine (T_4_), at an affinity ratio of 20-30-fold to 1, bind to the traditional thyroid hormone nuclear receptors (TRs), forming a ligand-TR complex [135]. Functionally, the T_3_-TR complex is the transcriptionally active form of thyroid hormone in the nucleus. T_4_ is viewed as a systemic prohormone, undergoing conversion to T_3_ as needed at the cell-tissue level. The T_3_-TRβ1 complex binds to promoter regions of thyroid hormone-responsive genes and regulates specific gene expression. T_3_-regulated genes via their gene product proteins control a large panel of normal biological activities [97, 98]. Additionally, thyroid hormone—primarily T_4_—can bind to plasma membrane integrin αvβ3 to stimulate cancer cell growth [98, 99]. The thyroxine-binding site on the integrin αvβ3 RGD binding domain is shown in Fig. 5. It topographically overlaps with a heteronemin postulated binding site on integrin αvβ3. The best binding model for heteronemin crosses through the thyroid hormone pocket, resveratrol pocket, and steroid pocket of the integrin; this model indicates that heteronemin not only competes for E_2,_ but also interferes with T_4_ binding. Evidence also indicates that thyroid hormones such as T_4_ not only stimulate cancer growth in hyperthyroid states but may also be done under euthyroid conditions. Thyroid hormone-induced ERK1/2 activation in tumor cells is correlated to the expression of PD-L1 by thyroid hormone [99]. Expression of PD-L1 links with inhibition of EGFR/ERK1/2 signaling cascades in cancer cells. Heteronemin combined with tetrac suppresses *PD-L1* expression. It may also be noted that KRAS status has an impact on the drug (heteronemin) efficacy in colorectal cancer cells [99].

In addition to promoting cancer cell proliferation, thyroid hormone T_4_ affects anti-cancer activities induced by different mechanisms by anticancer reagents [24]. Thyroxine induces PD-L1 that traps resveratrol-induced COX-2 in the cytoplasm to prevent its translocation into the nucleus to function as a co-activator [16]. Thyroxine also increases ribonucleotide reductase regulatory subunit M2 (RRM2) expression to reduce the effect of resveratrol-induced cancer cell growth. T_4_ activates P-glycoprotein (P-gp) that underlies multiple drug resistance in chemotherapies [100]. Since heteronemin blocks thyroxine activity, then P-gp efflux pumping is suppressed, which is similar to the action of T_4_ antagonist, tetrac. For this reason, heteronemin may increase the intracellular residence time of cancer chemotherapeutic agents and dismiss drug resistance.

Thyroid hormone stimulates EGFR expression in the mutant KRAS cells but the modulation of EGFR protein expression by thyroid hormone was not a significant change in HT-29 cells [14]. Moreover, EGFR cross talks with integrin αvβ3 signaling to activate ERK1/2 and *PD-L1* expression in mutant *KRAS* colorectal cancer (CRC) [14, 15]. EGFR tyrosine kinase inhibitor, gefitinib [14, 15], or an anti-EGFR monoclonal antibody, cetuximab [13], cannot inhibit the thyroid hormone-induced stimulatory effect of ERK1/2 activation and EGFR expression in *KRAS* mutant CRC. Hyperthyroidism status or even euthyroid status may show an inhibitory effect on heteronemin-induced anti-proliferation in xenograft or clinics.

**Fig. 6:**
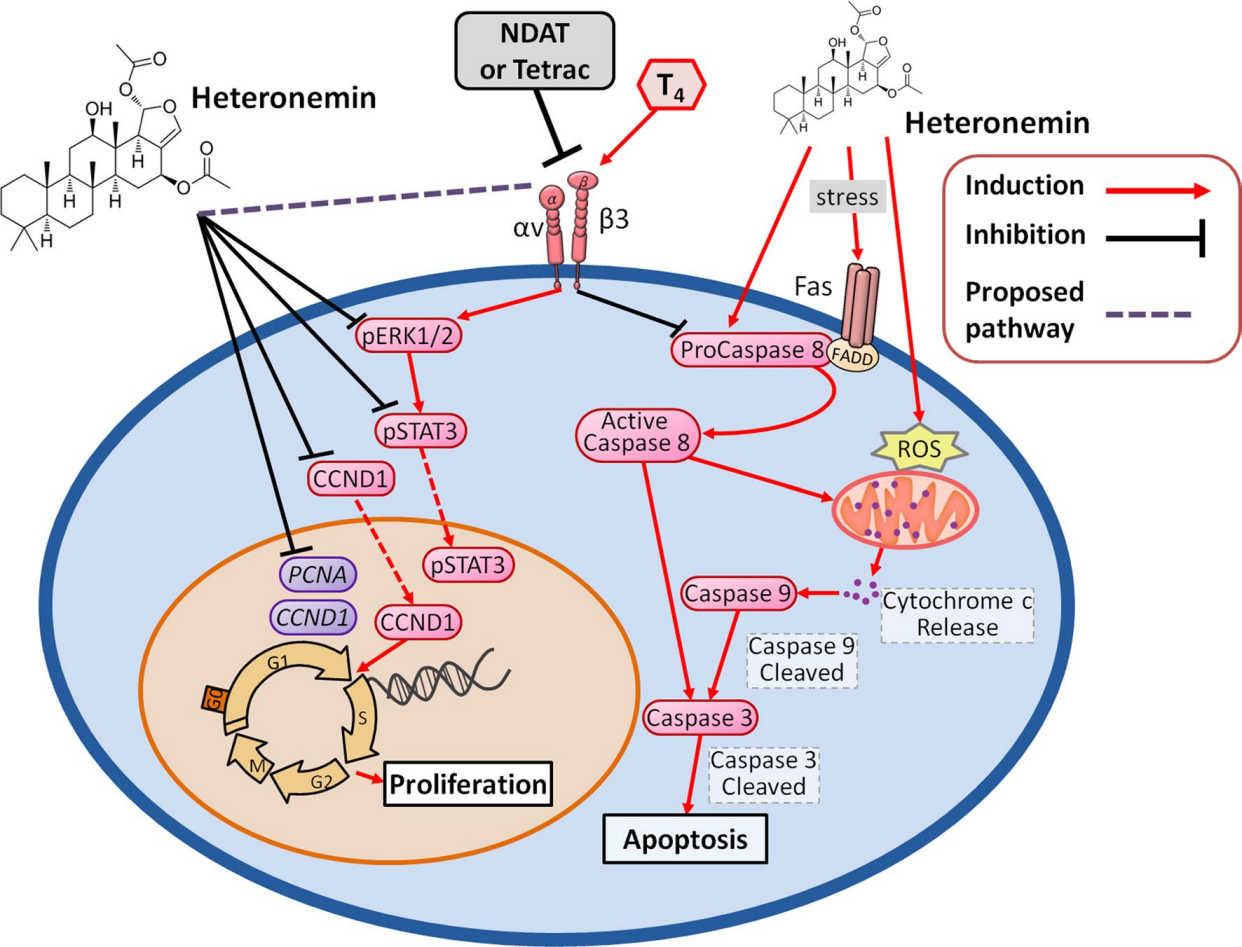
Signal transduction pathways are induced by thyroxine and heteronemin in cancer cells. Thyroxine binds to cell surface integrin αvβ3 receptor to activate signal transduction pathways such as ERK1/2 and STAT3. Those signals play vital roles in gene expression involved in cancer cell proliferation and metastasis. In addition, thyroxine suppresses pro-apoptotic gene expression and the activities of caspases. On the other hand, heteronemin suppresses ERK1/2 activation and increases ROS production. It inhibits the expression of proliferative genes. In addition, heteronemin activates the caspase signal pathway

Thyroid hormone stimulates ERK1/2 activation, STAT3 phosphorylation, and their downstream signal transduction pathways and related-gene expression. Conversely, tetraiodothyroacetic acid (tetrac), the deaminated analog of T_4_, and its nano-derivative (NDAT) suppress the ERK1/2 and STAT3 activation by blocking cell surface integrin αvβ3, with effective anti-proliferation against non-small-cell lung cancer (NSCLC) cells [12]. Similarly, heteronemin inhibits both ERK1/2 activation and STAT3 phosphorylation in lung cancer [12] and oral cancer [11] cells (Fig. 6). T_4_ has anti-apoptotic effects [101], however, and heteronemin induces apoptosis in human renal cancer [38], leukemia [18, 42], lung cancer [12], hepatocellular carcinoma [43]. Thyroxine also activates the expression of genes in cancer cells related to angiogenesis in cancer cells [102–104]. Consistent with these effects, T_4_ suppresses the expression of the X-linked inhibitor of apoptosis (*XIAP*, anti-apoptotic gene) and of anti-angiogenic thrombospondin 1 (THBS1) in human breast cancer cells [105]. Consistent with other effects of tetrac, the latter induces THBS1 in various cancer cells, including colorectal cancer, breast cancer, medullary carcinoma of the thyroid, and pancreatic cancer cells [105]. However, expression of *THBS-1* is induced by *TGF-β1* in cancer stroma and promotes invasion of oral squamous cell carcinoma [106]. Both heteronemin and tetrac inhibit *THBS-1* expression in oral cancer cells [11]. Combined treatment of such cells with tetrac and heteronemin inhibits *TGF-β1* expression almost entirely [11]. Such results indicate that thyroxine stimulates tumor cell proliferation and interferes with heteronemin-induced anti-proliferation. In other words, thyroxine-induced activity can be blocked by heteronemin, tetrac, or the combination of these agents.

Heteronemin downregulated the ERK/MAPK expression and also promoted the formation of ROS to activate antiproliferation in hepatocellular carcinoma cell lines [43]. Gene expression linked to colorectal cancer progression such as *EGFR, TGFB1, transforming growth factor-β receptor 2 (TGFBR2)*, and tumor protein 53 (*TP53*) and growth factor pathways were significantly reduced by combined treatment of heteronemin and tetrac [11]. The signaling transduction pathways involved in cancer progression in *KRAS* mutant cells have also been investigated. A number of signal pathways are up-regulated by heteronemin or combined treatment with heteronemin and tetrac.

### Heteronemin interacts with steroid hormones

Estrogen is generally regarded as a transcription factor in breast cancer cells that promotes proliferation and other important cancer cell survival functions via the nuclear estrogen receptor (ER) [107]. In ER-expressing breast cancers—such as the classical MCF-7 cell line—estrogen binds to the receptor to support breast cancer development. Of interest is that in MDA-MB-231 cells which are ER-negative, estrogen has a receptor to bind on the cell surface, namely, the ligand-binding site on plasma membrane integrin αvβ3 [90]. However, the intracellular signaling mechanisms downstream of the integrin receptor for estrogen require further characterization. Substantial evidence indicates that oxidative metabolism of estrogens may also play an important role in initiating breast cancer development [108, 109]. 2-Hydroxyestradiol (2OHE_2_) and 4-hydroxyestradiol (4OHE_2_), two metabolites of estrogen, are greatly redox-active and generate ROS in breast epithelial cells [110]. ER-mediated signaling plays a vital role in breast cancer growth but apparently does not play a role in cancer initiation [111]. Estrogen opposes stilbene(resveratrol)-induced antiproliferation [112]. Heteronemin inhibits cell proliferation in both ER-positive MCF-7 and ER-negative MDA-MB-231 cells and the effects are partially reversed by estrogen [10], consistent with effects initiated at the integrin receptor for the steroid, as discussed next.

**Fig. 7:**
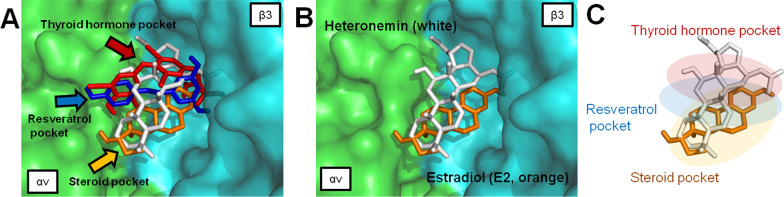
The best binding mode of heteronemin is superimposed with estradiol. Thyroxine (T_4_), resveratrol (RSV), estradiol (E_2_), and heteronemin bind into integrin αvβ3, and were performed in red, blue, orange, and white, respectively. **A** T_4_ (red), RSV (blue) and E_2_ (orange) binding mode represent the thyroid hormone pocket, resveratrol pocket, and steroid pocket, respectively. **B** The best-docked mode of heteronemin maps with the E_2_. **C** Heteronemin prefers to occupy the binding sites in a part of the steroid pocket

Estrogen (estradiol, E_2_) has been shown to interact with a receptor on plasma membrane structural protein integrin αvβ3 and to activate ERK1/2, which downstream may interact with nuclear ERα [93]. Since the non-peptide hormone E_2_ reverses the anticancer effects induced by heteronemin, the latter may inhibit cancer proliferation through multiple discrete mechanisms related to cell surface integrin αvβ3. The postulated interaction between E_2_ and heteronemin on integrin αvβ3 is shown in Fig. 7. Heteronemin occupies a part of the steroid pocket and thus interferes or competes with the binding of E_2_ to αvβ3 (Fig. 7C). For that reason, heteronemin-induced anti-proliferation is partially blocked by estrogen in MCF-7 cells. On the other hand, estrogen does not stimulate cell growth in ERα-deficient MDA-MB-231 cells and only minimally reverses heteronemin-induced antiproliferation in these cells [10].

Estrogens in physiologically available concentrations or estrogen metabolites directly act on the mitochondria of mammary epithelial cells to induce the production of ROS [110]. The increasing ROS subsequently enhances different kinase activities that have effects on redox-sensitive transcription factors [113, 114]. Estrogen promotes a large accumulation of ROS that can play a key role in driving carcinogenesis [115]. Excessive ROS increases genomic instability and activates the redox-associated signaling pathway and ROS thus serves an important role in estrogen-induced cancer.

Estrogen stimulates phosphorylation of ERK1/2, protein kinase C alpha (PKCα), and STAT3 [10]. Such activation of signal transduction may relate to the generation of ROS [110]. In contrast, heteronemin—in the absence of estradiol—inhibits the activation of ERK1/2 and STAT3 [10], but can stimulate PKCα phosphorylation in MCF-7 cancer cells. When estrogen is present, heteronemin reverses the sex steroid's induction of phosphorylation of ERK1/2 and STAT3. Sotrastaurin (SOT), a PKC inhibitor, blocks the activation of ERK1/2, PKCα, and STAT3 in MCF-7 cells. The inhibitory effect of SOT on the activities of ERK1/2 and STAT3 is further enhanced by heteronemin treatment [10]. As expected, estrogen does not affect STAT3, PKCα, or ERK1/2 phosphorylation in MDA-MB-231 cells. Heteronemin at 0.3125 µM inhibits the phosphorylation of STAT3 and ERK1/2, but slightly increases PKCα activation in the absence of ER, i.e., in MDA-MB-231 cells. However, the inhibitory effect of heteronemin on pSTAT3 is blocked by estrogen. Activated PKC has been shown to support the survival of MDA-MB-231 cells [116]. SOT only inhibits PKC activity, but not the actions of ERK1/2 and STAT3 in MDA-MB-231 cells. Such results suggest that the roles of PKC in MDA-MB-231 cells differ from those in MCF-7 cells.

Estrogen significantly increases the expression of *PCNA*, *CCND1*, *EGFR*, and *PD-L1* in ER-positive breast cancer MCF-7 cells [10]. Heteronemin inhibits a number of estrogen-induced stimulatory effects on gene expression. Although 0.625 µM heteronemin does not inhibit *CCND1* expression, it does reverse estrogen-induced *CCND1* expression in ER-positive breast cancer cells. On the other hand, in ER-negative MDA-MB-231 cells, estrogen did not affect the expression of *PCNA*, *CCND1*, *EGFR*, and *PD-L1*. However, co-treatment with heteronemin and estrogen did not permit estrogen-induced expression of *PCNA*, *EGFR*, and *PD-L1* [10].

**Fig. 8:**
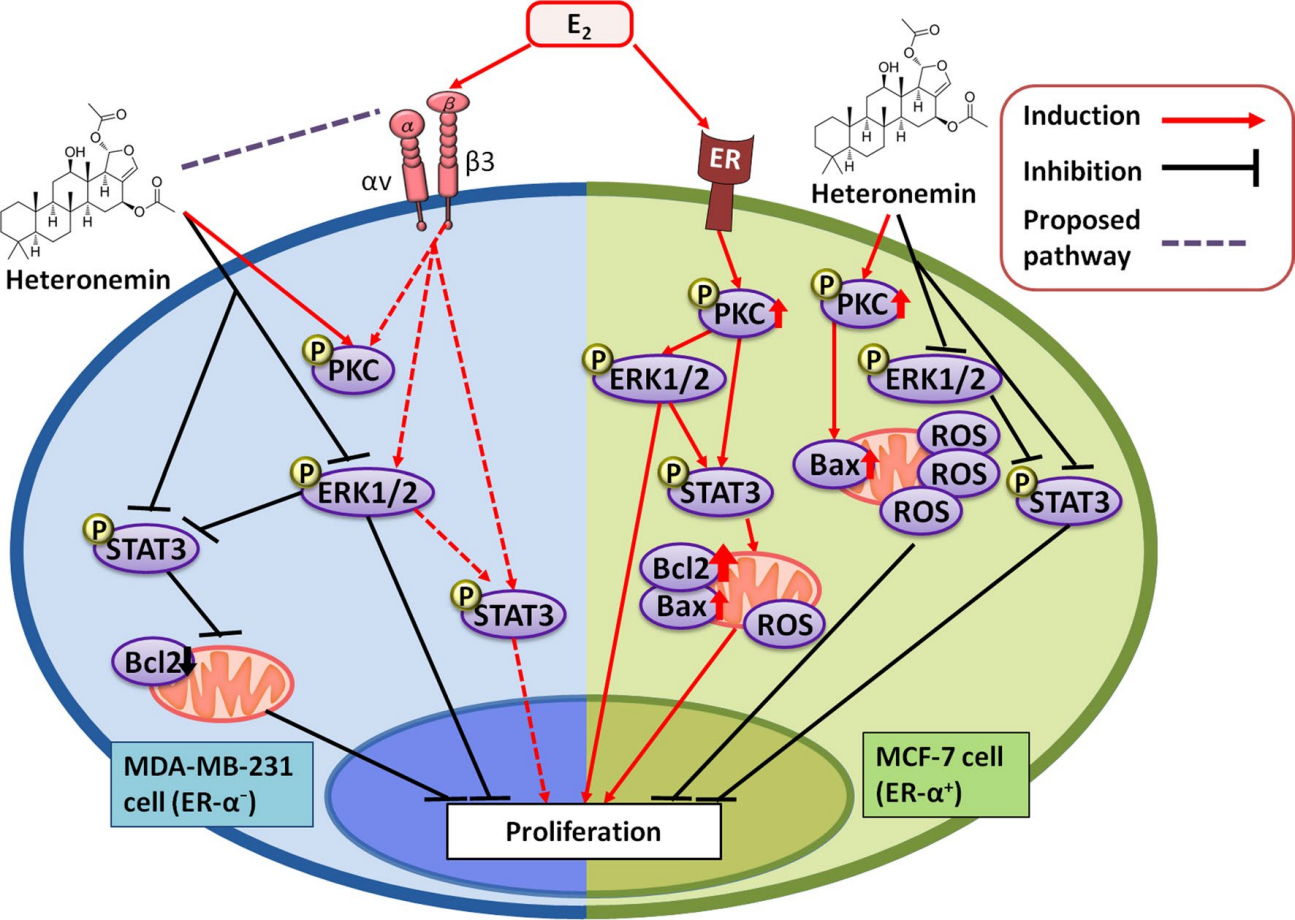
Signal transduction pathways induced by estrogen and heteronemin in ER-positive and -negative breast cancer cells. Estrogen binds to ER-α at the cell surface or integrin αvβ3 receptor in ER-positive or ER-negative breast cancer cells, respectively to activate signal transduction pathways such as ERK1/2 and STAT3. Those signals play vital roles in gene expression involved in cancer cell proliferation and metastasis. In addition. On the other hand, heteronemin suppresses ERK1/2 and STAT3 activation and inhibits the expression of proliferative genes. In addition, heteronemin activates the caspase signal pathway

An increase in the Bcl-2:Bax ratio reflects cancer cell proliferation, while a decrease accompanies apoptosis [117]. In addition to studies of the Bcl-2:Bax ratio, we have examined the expression of Bax, BAD, and p21 in response to estrogen and heteronemin. In MCF-7 cells, estrogen increases the expression of Bax and the Bcl-2:Bax ratio, but inhibits the expression of important anti-proliferative genes, BAD and p21. Heteronemin increases the expression of Bax, BAD, and p21 but estrogen abolished this pro-apoptotic effect. The Bcl-2:Bax ratio increases in heteronemin-treated cells in the presence of estrogen. In contrast, estrogen does not affect the expression of Bax, BAD, and p21 in MDA-MB-231 cells. Heteronemin inhibits the expression of Bax, but significantly stimulates the expression of BAD and p21. It also reduces the Bcl-2:Bax ratio. In the presence of estrogen, the Bcl-2:Bax ratio is significantly inhibited, but the expression of BAD and p21 increases. In ER-positive MCF-7 cells, estrogen stimulates the expression of proliferation-related genes and inhibits the expression of pro-apoptotic genes [10], as noted above and summarized in Fig. 8.

Estrogen increases the expression of TGF-β1 which may promote the production of ROS [118]. In contrast, heteronemin has a biphasic effect, stimulating the expression of TGF-β1 at 0.625 µM, but inhibiting its expression at 1.25 µM. The stimulating effect of estrogen on TGF-β1 is inhibited by heteronemin treatment in MCF-7 cells [10]. Therapeutic targeting of mitochondrial uncoupling protein 2 (UCP2), with the resultant enhancement of ROS production, may increase cancer cell susceptibility to apoptosis [119]. UCP2 expression is inhibited by heteronemin in a concentration-dependent manner. Interestingly, estrogen also increases the production of mitochondrial ROS.

Overall, estrogen extends the cell cycle S phase and G2/M phase, but reduces G0/G1 phase. Conversely, heteronemin treatment increases G0/G1 phase population but decreases the S phase [10]. Co-treatment of estrogen and heteronemin decreases G0/G1 phase and increases the S phase. Heteronemin induces G0/G1 arrest and then reduces cell proliferation. In addition, heteronemin can reverse the cell cycle distribution induced by estrogen. SOT, an inhibitor of PKC, enhances the G0/G1 phase accumulation and the decreasing by heteronemin of the S phase. Similar results were obtained in MDA-MB-231 cells except that estrogen treatment did not affect the cell cycle.

Previously, our groups have demonstrated that dihydrotestosterone (DHT) and its target nuclear androgen receptor (AR) are also involved in interactions with cell surface integrin αvβ3 [93]. Andersen et al. [120] recently demonstrated that the marine sponge-derived small molecule EPI-001 inhibits both the androgen-dependent and androgen-independent activation of AR. Furthermore, Hoy et al., discovered that compounds isolated from marine organisms target the androgen receptor (AR) amino-terminus [121]. EPI-001 covalently binds to the AR-activation function-1 (AF-1) region of the N-terminal domain [122]. The EPI compound can inhibit the transcriptional activity of AR, as well as the transcriptional activity of a truncated form of the AR that lacks the ligand-binding domain. EPI-001 did not interfere with ligand binding. However, the agent blocks the androgen-dependent N/C interaction. The interaction is required for ligand-mediated activation of AR. The interactions between AR and trans-activating proteins are blocked by EPI-001 to inhibit androgen-induced proliferation. EPI-001 is non-toxic in vivo [120]. However, the effect of androgen on heteronemin-induced antiproliferation has not been fully determined.

### Heteronemin interacts with growth factors

Epidermal growth factor receptors (EGFRs) are expressed on the surface of normal, nonmalignant cells, and overexpressed by cancer cells. The ligands of EGFR are epidermal growth factor (EGF), TGF-β, amphiregulin, and epiregulin [123]. Downstream of EGFR, the two principal signal transuding pathways are the PI3K/AKT/mTOR and RAS/RAF/MAPK cascades [123, 124]. However, the signal transducer and activator of the transcription 3 (STAT3) pathway also respond to EGFR signaling [123, 124] and the insulin-like growth factor 1 (IGF-1) receptor can dimerize with EGFR and activate EGFR signaling. In addition, downstream crosstalk between or among signaling cascades may occur. EGFR activation in cancer is associated with cancer progression. After ligand activation, EGFR phosphorylates and activates the RAS/RAF/MAPK, PI3K/AKT, and STAT/JAK pathways [123, 124]. Consequently, transcription factors are engaged and modulation may occur of the cell cycle, or angiogenesis or apoptosis may result. Activation of the EGFR signal transduction pathway may also induce cell motility [125]. EGFR-mediated signaling in mutant *KRAS* cells may include roles for EGFR/RAS/ERK or EGFR/RAS/PI3K pathways in cell proliferation and angiogenesis [126]. Signaling systems downstream of EGFR may also include JAK2, Src, and PI3 K/AKT, further activating STAT3 through phosphorylation at the Src homology 2 (SH2) domains [127]. Phosphorylated STAT3 proteins form dimers and translocate to the nucleus, where they may be involved in regulating the expression of proliferation-relevant genes, such as Cyclin D1, c-Myc, p53, and p21. EGF also modulates the activation of survival genes such as Bcl-2, Bcl-xL, Mcl-1, and Survivin. EGF also regulates the expression of MMP-2 and MMP-9 in metastasis and VEGF in angiogenesis [127, 128]. Blockade of the STAT3 signaling pathway may induce apoptosis and has been shown to eradicate tumor-initiating cells related to prostate cancer [129, 130]. EGF interaction with EGFR has also been shown to involve crosstalk with integrin αvβ3 [87, 88].

Anti-EGFR monoclonal antibodies, such as cetuximab and panitumumab, and several tyrosine kinase inhibitors (TKIs) that are active at EGFR are examples of signaling target therapies. However, these drugs are ineffective for patients with *KRAS* or *BRAF* mutations. Thus, their therapeutic efficacy depends on the genetic profile of the cancer patients (wild-type or mutant *KRAS* and *BRAF*) [131]. Heteronemin has been shown to inhibit EGFR activity [10, 12]. It enhances cytarabine-induced antiproliferation activity by blocking Ras protein farnesylation, leading to decreased activity of MAPK, AP-1, NF-κB, and c-myc [65]. Heteronemin down-regulates the expression level of the EGFR gene involved in cell movement [10, 12]. On the other hand, the deaminated thyroxine analogue, tetrac, displays anticancer effects that include decreased cell division and tumor growth, increased success ability to apoptosis and abrogated tumor-linked angiogenesis [132]. Treatment with heteronemin or its combination with tetrac, has been shown to down-regulate the gene expression level of EGFR [10], STAT3, and genes involved in tumor cell movement [10–12]. Pathway scores for cell proliferation, cell growth factors, cell differentiation, choline cancer metabolism, and carbon cancer metabolism have been shown to correlate with the functional status of EGFR.

Hepatocyte growth factor (HGF) activated signaling plays a vital role in prostate cancer development. HGF-activated STAT3 has been shown to link to the HGF/c-MET axis to stimulate the progression of metastatic prostate cancer [133, 134]. The marine natural product heteronemin exhibits potent antitumor effects by inhibiting c-Met/STAT3 activation in HGF-stimulated refractory prostate cancer cells [19]. An earlier molecular docking study suggested that heteronemin is a potent STAT3 inhibitor, acting via the STAT3 SH2 domain [19]. Additionally, Chen et al. have demonstrated that heteronemin can target the receptor cavities of VEGF-C/VEGFR-3, thereby hindering the phosphorylation of VEGFR-3 [20]. Heteronemin suppresses TGF-β expression and TGF-β pathway-dependent cancer cell proliferation [44]. This marine product also inhibits TGF-β expression and the growth factor’s effect on antiproliferation, anti-migration, and anti-adhesion effects [44]. Heteronemin efficiently inhibited the HGF-stimulated c-Met/STAT3 [19, 44] in prostate cancer cells [19] and also suppressed HGF-induced colony formation [19]. Other cellular processes affected by heteronemin in TNF-α-treated cells include the cell cycle, apoptosis, the MAPK pathway and the NF-κB signaling cascade [42]. Thus, there is a substantial interaction effect between heteronemin and TNF-α [42].

**Fig. 9:**
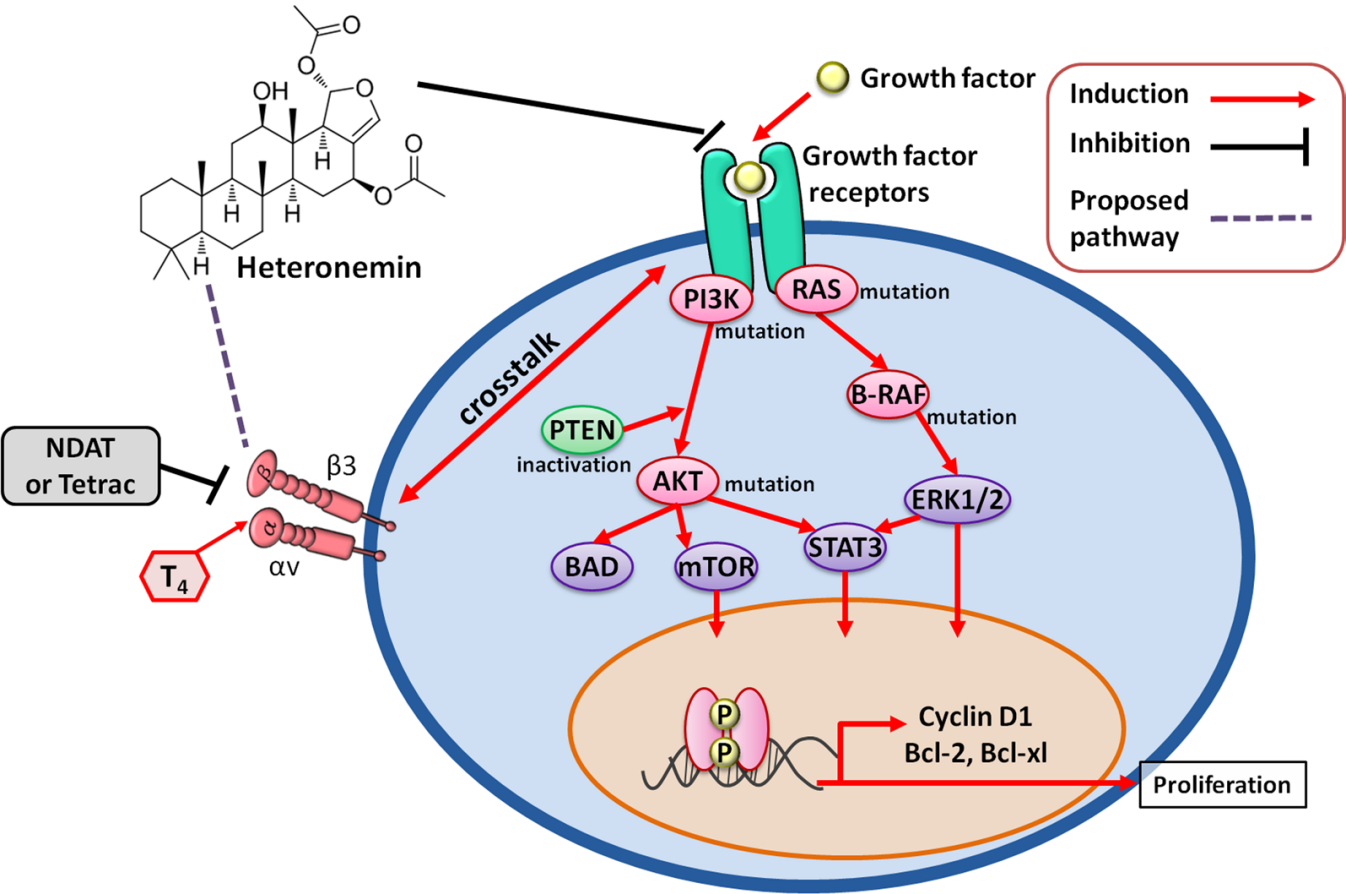
Effect of growth factors on heteronemin-induced anti-proliferation in human cancer cells. Growth factors bind with particular receptors and stimulate signal transduction pathways, mainly PI3K and ERK1/2. In addition, STAT3 activation involves cancer cell proliferation. The signals of growth factors may engage in crosstalk with integrin αvβ3. The working model for heteronemin suppresses the tumorigenicity of cancer cells through the inactivation of PI3K, ERK1/2, and STAT3. Heteronemin perhaps not only inhibited downstream of integrin αvβ3, ERK/MAPK pathway, but also crosstalk with growth factor receptor to suppress the PI3K/AKT/mTOR pathway

Noted earlier in this review is that there is cross-talk between thyroid hormone-integrin αvβ3 signaling and EGFR [87–89]. Suppression of integrin αvβ3 results in inhibition of EGFR sialylation induced by β-Galactoside α-2,6-sialyltransferase 1 (ST6Gal1) [14]. Additionally, gefitinib inhibits cell proliferation in wild-type *KRAS* HT-29 cells, and tetrac-based NDAT significantly enhances the gefitinib-induced antiproliferation [14]. Conversely, gefitinib inhibits cell proliferation in mutant *KRAS* HCT-116 cells only in high concentrations; this effect is further enhanced by NDAT [14, 89]. We can therefore conclude that thyroid hormone may interfere with EGFR-regulated signaling (Fig. 9). Because heteronemin may disturb the activities of integrin αvβ3, combined treatments with heteronemin and other anticancer agents, such as tetrac (or NDAT) or growth factor receptor inhibitors, may have a synergistic therapeutic effect.

The synergies of heteronemin with other anticancer agents are complex. For example, heteronemin inhibits VEGFR-3 phosphorylation and reduces downstream phosphorylation of MEK/ERK and NF-κB [20]. In A-498 cells, the agent has also been shown to inhibit PI3K/AKT and ERK1/2 pathways downstream of EGFR and induce apoptosis via activation of caspase-3, caspase-8, and caspase-9 [38]. Then the combination of heteronemin and tetrac (or NDAT) downregulates EGFR/STAT3 expressions and cell motility in *KRAS* mutated A549 non-small cell lung cancer (NSCLC) cells [12]. Tetrac and NDAT also effectively inactivate the phosphorylation of the VEGFR and ERK1/2 signaling pathway and improve heteronemin-induced antiproliferative activity in *KRAS* mutant NSCLC cells [12]. Heteronemin or tetrac (or NDAT) and their combination significantly decrease the expression of *STAT3* and *VEGFR* mRNA in A549 cells. Both heteronemin and tetrac or NDAT inhibit ERK1/2 activation and STAT3 phosphorylation in A549 cells [12]. These observations are consistent with the fact that the suppression of ERK1/2 or PI3K activation can inhibit STAT3 phosphorylation.

## Conclusions and summary remarks

Marine natural products are a source of molecular structures with potentially important biological/pharmacological actions in clinical contexts. Heteronemin, isolated from sponges, inhibits cancer growth via different mechanisms. The anticancer activity of the compound depends in part on the disruption of several signal transduction pathways. Heteronemin inhibits ERK1/2, PI3K, STAT3, and NF-κB activation in several types of human cancer cells. Heteronemin also inhibits the expression of proinflammatory cytokines, such as IL-1 and TNF-α, which are involved in the pathogenesis of cancer development. The interactions between hormones, growth factors, and anticancer drugs continue to be extensively studied. In the context of cancer cell biology, hormones are known to affect tumor cell proliferation, metastasis, and invasiveness. Growth factors bind cell surface receptors to activate downstream signal pathways in cancer cells, as they do in nonmalignant cells. The nonpeptide hormones have different receptors to activate signal transduction and may each bind to a discrete receptor on integrin αvβ3 to induce ERK1/2 activation. Growth factors also activate ERK1/2. Downstream of activated ERK1/2, nonpeptide hormones, and growth factors interfere with heteronemin-induced antiproliferation action in tumor cells. Although heteronemin down-regulates ERK1/2 activation, heteronemin-induced antiproliferative activities oppose the physiologic concentrations of nonpeptide hormones and growth factors. In this review, we have emphasized the role that heteronemin plays in cancers and its interaction with thyroid hormones, steroid hormones, and growth factors. Schemas of the signal transduction pathways by heteronemin and different nonpeptide hormones are presented. We hypothesize that physiological circulating levels of hormones and growth factors may interfere with heteronemin-induced anticancer activities in the xenograft animal model and the clinic. The clinical efficacy of heteronemin in the therapy of cancer may be potentiated by inhibiting the cell surface receptors, such as integrin and EGFR, for nonpeptide hormone, as well as receptors for several other endogenous factors that are able to suppress the antiproliferation activity of heteronemin. Molecular modeling results revealed that heteronemin occupied the thyroid hormone pocket, resveratrol pocket, and steroid pocket of integrin αvβ3. This implies that heteronemin can influence the effect of thyroxine, stilbene-like agents (resveratrol), and steroid skeleton molecules (estrogen, progesterone, and androgen) to modulate consequent biological functions. All of this evidence suggests that heteronemin can be used to reduce cancer-related inflammation and cancer growth. The combination of tetrac and heteronemin might potentially be used as an alternative strategy to make it more effective in *KRAS* mutations acquired resistance to anti-EGFR therapy. However, more preclinical and clinical studies are required to determine the safety and effectiveness of this combination. In conclusion, this review contributes to a comprehension of the potential of the heteronemin and accelerates the development of its new applications in drug discovery.

## Data Availability

Not applicable.

## Abbreviations

AF-1: AR-activation function-1
AP-1: Activator protein-1
AR: Androgen receptor
Bcl-2: B-Cell Lymphoma 2
CCND1: Cell cyclin D1
CRC: Colorectal cancer
DHT: Dihydrotestosterone
E_2_: Estradiol
ECM: Extracellular matrix
EGF: Epidermal growth factor
EGFR: Epidermal growth factor receptor
ER: Estrogen receptor
ERK: Extracellular signal-regulated kinase
5-FU: 5-Fluorouracil
FAK: Focal adhesion kinase
FDA: Food and Drug Administration
FTI: Farnesyl transferase inhibitor
Gpx4: Glutathione peroxidase 4
HCC: Hepatocellular carcinoma
HGF: Hepatocyte growth factor
IGF-1: Insulin-like growth factor-1 (IGF-1)
JNKs: C-Jun N-terminal kinases
KDAC: Lysine deacetylases
MAPK: Mitogen-activated protein kinase
MMP: Matrix metalloproteinase
NAC: *N*-Acetyl cysteine
NDAT: Nano-diamino-tetrac
NF-κB: Nuclear factor kappa B
NSCLC: Non-small-cell lung cancer
PCNA: Proliferating cell nuclear antigen
PD-L1: Programmed death-ligand 1
P-gp: P-Glycoprotein
PKCα: Protein kinase C alpha
RGD: Arginine-glycine-aspartate
ROS: Oxygen species
RRM2: Ribonucleotide reductase regulatory subunit M2
RSV: Resveratrol
SH2: SRC homology 2
SMAD: Mothers against decapentaplegic homolog
SOD: Superoxide dismutase
SOT: Sotrastaurin
STAT3: Signal transducer and activator of transcription 3
T_3_: Triiodothyronine
T_4_: *L*-Thyroxine
TDP-43: Trans-activation response DNA-binding protein of 43 kDa
Tetrac: T_4_ac, Tetraiodothyroacetic acid
TGF-β: Transforming growth factor-beta
THBS-1: Thrombospondin-1
TKI: Tyrosine kinase inhibitor
TNFα: Tumor necrosis factor alpha
TRβ1: Thyroid hormone receptor β1
UCP2: Uncoupled protein 2
XIAP: X-Linked inhibitor of apoptosis
